# Physiological Responses of Juvenile Bullfrogs (*Aquarana catesbeiana*) to Salinity Stress

**DOI:** 10.3390/ani14233454

**Published:** 2024-11-29

**Authors:** Xiaoting Zheng, Xueying Liang, Qiuyu Chen, Jingyi Xie, Hongbiao Dong, Jinlong Yang, Jiasong Zhang

**Affiliations:** 1 Key Laboratory of South China Sea Fishery Resources Exploitation & Utilization, Ministry of Agriculture and Rural Affairs, South China Sea Fisheries Research Institute, Chinese Academy of Fishery Sciences, Guangzhou 510300, China; xtzheng1990@163.com (X.Z.); changxun100@163.com (X.L.); ciiyii1010@163.com (Q.C.); 18699664918@163.com (J.X.); donghongbiao@163.com (H.D.); 2 College of Fisheries and Life Science, Shanghai Ocean University, Shanghai 201306, China; jlyang@shou.edu.cn; 3 Sanya Tropical Fisheries Research Institute, Sanya 572018, China

**Keywords:** bullfrog, salinity stress, survival rate, digestive enzyme, antioxidant enzyme

## Abstract

Bullfrogs (*Aquarana catesbeiana*) are increasingly farmed for their high nutritional value and adaptability to intensive aquaculture systems. However, salinity stress due to environmental changes and habitat salinization poses a significant challenge for both wild and farmed bullfrogs. This study examines the physiological responses of juvenile bullfrogs to varying salinity levels (0, 2, 4, 6 ppt) to better understand their capacity for osmoregulation and adaptation to salinization. Juvenile bullfrogs underwent salinity treatments for one week, and various physiological parameters, including digestive enzyme activity, antioxidant enzyme activity, and serum biochemical indicators, were measured. The study revealed that moderate salinity (2–4 ppt) enhanced pepsin and amylase activity while maintaining high survival rates. However, higher salinity levels (6 ppt) impaired antioxidant defense mechanisms and liver tissue, increasing oxidative stress markers such as malondialdehyde (MDA). The results suggest that bullfrogs possess a degree of salt tolerance, which may predict their resilience to future landscape salinization driven by environmental changes. This research provides valuable insights into the osmoregulatory mechanisms of amphibians under salinity stress, addressing a critical gap in knowledge essential for both conservation and aquaculture management.

## Simple Summary

Environmental changes leading to increased salinity levels pose significant challenges to freshwater species. This study explores the physiological mechanisms by which juvenile bullfrogs cope with varying salinity conditions, offering new insights into their potential resilience in salinizing freshwater ecosystems. While previous research has largely focused on the early developmental stages, such as eggs and tadpoles, this study fills a crucial gap by examining juvenile bullfrogs. By evaluating key physiological parameters, including digestive enzyme activity, antioxidant responses, and serum biochemical markers, we uncover important aspects of their osmoregulatory capacity. These findings have implications for enhancing aquaculture practices and supporting conservation strategies, contributing to the sustainable management of bullfrog populations under shifting environmental conditions.

## 1. Introduction

With the escalation of freshwater ecosystem salinization due to climate change, agricultural runoff, and urbanization, understanding how salinity affects these environments has become a pressing global ecological challenge [1]. Freshwater salinization leads to both lethal and sublethal effects on aquatic organisms, impacting their growth, reproduction, and overall health [2,3]. These effects cause changes in the structure and functioning of freshwater ecosystems [4]. Despite the growing prevalence of freshwater salinization worldwide, research on organismal strategies to mitigate saline stress remains nascent. Amphibians, with their highly permeable skin and limited osmoregulatory capacity, offer an excellent model for studying the effects of elevated salinity [5]. Their physiological characteristics make them particularly vulnerable to osmotic stress, as they struggle to concentrate and excrete excess salts, resulting in reduced survival, growth retardation, and developmental delays [6,7]. This study focuses on the physiological responses of American juvenile bullfrogs (*Aquarana catesbeiana*) to saline conditions.

The American bullfrog (*A. catesbeiana*), originally native to North America, is one of the largest and most widely distributed frog species globally [8]. Renowned for its distinctive deep vocalizations, this species is of ecological, economic, and biological importance [9,10,11]. It also serves as an ideal model for studying the effects of salinity stress on juvenile amphibians. Most previous research has focused on the early developmental stages, such as eggs and tadpoles, which are particularly vulnerable to osmotic stress. For instance, bullfrog eggs and tadpoles younger than 10 days old experience 100% mortality when exposed to a salinity of 6 ppt for 10 days. Similarly, tadpoles aged 10–15 days exhibit markedly decreased survival rates under the same salinity conditions, underscoring their heightened sensitivity during early development [12]. These stages are especially susceptible because they occur in aquatic environments where water salinity directly affects osmoregulation, growth, and development [7,13,14].

However, there is a notable gap in the literature regarding juvenile frogs’ salinity tolerance, a stage crucial for their transition to terrestrial environments and overall survival. Unlike eggs and tadpoles, juvenile frogs are semi-aquatic, meaning they are exposed to salinity stress in both aquatic and terrestrial environments [15]. As amphibians mature, their osmoregulatory systems become more sophisticated, allowing them to manage salinity stress more effectively than at earlier developmental stages [16,17]. Despite this, little research has been conducted on the physiological mechanisms and adaptive responses of juvenile bullfrogs to salinity stress.

This research addresses this gap by investigating how varying salinity levels affect the physiological and biochemical responses of juvenile bullfrogs. By focusing on this life stage, we seek to offer insights into how juvenile frogs, and potentially other amphibians, will adapt to the increasing salinization of freshwater ecosystems, a process exacerbated by climate change and drought.

## 2. Materials and Methods

### 2.1. Animal and Acclimation

The juvenile bullfrogs were obtained from a commercial farm (Huizhou Boluo Wubo Animal Husbandry and Fisheries Co., Ltd., Huizhou, China). Bullfrogs were selected based on the health status of individuals, namely consistent specifications, robust physique, and the absence of disease or injury. This experiment was conducted at the Zhuhai Experimental Base of the South China Sea Fisheries Research Institute, Chinese Academy of Fishery Sciences (Zhuhai, China). The juvenile bullfrogs were acclimated for one week under laboratory conditions in a 250 × 148 × 102 cm canvas pool filled with continuously aerated tap water (10 cm depth, temperature 24.5 ± 0.5 °C; pH 7.1 ± 0.3; 11L:13D light/dark cycle). During the acclimatization period, the bullfrogs were fed commercial feed (SKRETTING biotechnology Co., Ltd. (Zhuhai, China)), containing crude protein ≥ 42.0%, moisture ≤ 10.0, crude fat ≥ 4.0%, crude fiber ≤ 4.0%, effective lysine ≥ 2.20, crude ash ≤ 15.0, total phosphorus ≥ 1.20, calcium ≤ 4.50, and excrement and food residues were regularly removed.

### 2.2. Experimental Design

Following acclimatization, 180 bullfrogs (body weight 32.19 ± 7.60 g, body length 7.33 ± 0.42 cm) were randomly divided into 12 tanks (four treatment groups with three replicate tanks) (43 × 33 × 50 cm with 10 cm water depth) at a density of 15 bullfrogs per tank. The four treatment groups consisted of different salinity levels, namely 0 (control), 2, 4, and 6 ppt. The 0 ppt group was dechlorinated tap water, while the salinities of the other three were raised to 2, 4, and 6 ppt by adding commercial sea salt mixes (Jiangxi Haiding Technology Co., Ltd., Ji’an, China). The experiments were conducted in an indoor environment, with feedings administered twice daily at 8:30 and 18:30, and excrement and food residues were regularly removed. The environmental conditions during the experiment were consistent with those during the acclimatization period, with daily water changes (100%). The experimental duration was one week.

### 2.3. Sample Collection and Analysis

Bullfrogs were starved for 24 h prior to sampling at the end of the experiment. The total number of bullfrogs in each tank was recorded, and seven bullfrogs per tank were randomly selected for anesthesia using the double-destruction medullary method. Blood was collected from the hearts of the seven bullfrogs using a 1-mL syringe and allowed to clot at room temperature for one hour to obtain serum samples by centrifugation (1500× *g*, 10 min, 4 °C) and stored at −80 °C until analysis. After blood collection, the livers and intestines of the seven bullfrogs were sequentially sampled. Three bullfrogs’ liver and intestine samples were collected and fixed in 4% formaldehyde for histological examination, while the remaining four bullfrogs’ liver and intestine samples were stored at −80 °C until analysis. All sample collection processes were performed on ice.

The procedures for collecting and handling the animals adhered strictly to the guidelines of the Institution Animal Care and Use Committee of the South China Sea Fisheries Research Institute (SCSFRI-CAFS, No. nhdf2024-16) and complied with China’s Animal Welfare Legislation.

### 2.4. Histological Examination

Liver (5 mm × 3 mm × 3 mm) and intestinal tissue (5 mm length) samples were sent to Wuhan Sevier Biotechnology Co., Ltd. (Wuhan, China) for embedding and paraffin sectioning. The specific steps included washing with running water, dehydration with graded alcohol, clearing in xylene, embedding in paraffin, sectioning at 4 μm, staining with hematoxylin and eosin, and observing and collecting images under a light microscope [8]. In addition, liver tissue was also stained with PAS and Oil Red O to observe polysaccharide and lipid content [18].

### 2.5. Digestive Enzyme Analysis

The intestine samples were homogenized in 10 volumes (*w*/*v*) with ice-cold sterile normal saline solution (0.86% NaCl) and centrifuged at 4 °C (1000× *g*, 10 min) on ice. Activities of amylase and pepsin were analyzed using commercial assay kits (Nanjing Jiancheng Institute of Biological Engineering, Nanjing, China) according to the manufacturer’s instructions [19]. Additionally, protein concentrations were determined using commercial assay kits (Nanjing Jiancheng Bioengineering Institute, Nanjing, China) according to the manufacturer’s instructions [19]. Enzyme activities were quantified as units per milligram of protein.

### 2.6. Serum Biochemical Analysis

The activities of blood urea nitrogen (BUN), glucose (GLU), lactate dehydrogenase (LDH), ammonia (NH3), creatinine (CRE), and cortisol (COR) in the serum were determined using commercial kits (Nanjing Jiancheng Institute of Biological Engineering, Nanjing, China) according to the manufacturer’s instructions [20].

### 2.7. Antioxidant Enzyme Activities Analysis

According to the kit instructions, intestinal and liver samples were weighed separately and homogenized by adding nine times their volume of PBS buffer solution (1:9, g·mL⁻¹). The homogenized samples were maintained at 4 °C, followed by centrifugation at 3000 rpm for 10 min. The supernatant was collected to measure antioxidant indicators such as total antioxidant capacity (T-AOC), superoxide dismutase (SOD), catalase (CAT), and malondialdehyde (MDA) in both the intestine and liver [8]. All indicators were measured using a multifunctional microplate reader with kits supplied by Nanjing Jiancheng Bioengineering Institute (Nanjing, China).

### 2.8. Statistical Analysis

The data were presented as the mean ± standard deviation, and enzyme activity data were transformed to meet the assumptions of normality and homoscedasticity as needed. The statistical analysis was performed using one-way ANOVA (SPSS for Windows, Version 22.0), followed by post hoc Duncan multiple range tests to determine significant differences between the four salinity groups. A *p*-value of < 0.05 was considered statistically significant. The results were presented using GraphPad Prism software (version 7, GraphPad Software, Inc., San Diego, CA, USA), including all biological replicates and levels of significance.

## 3. Results

### 3.1. Effect of Salinity on the Survival of Juvenile Bullfrogs

In all experimental variants the survival was 100% (Table 1).

### 3.2. Effects of Salinity on the Intestinal and Liver Morphology of Juvenile Bullfrogs

**Figure 1:**
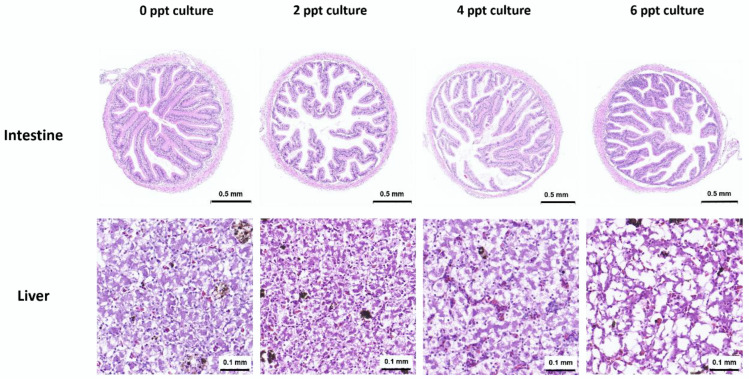
Intestinal and liver morphology of juvenile bullfrogs (*A. catesbeiana*) under the light microscope after different salinity level culture for one week.

In the control group (0 ppt), the intestinal mucosal structure remained intact, with villus clusters displaying a regular and organized structure. The height and width of the intestinal mucosal villi, as well as the thickness of the muscle layer, were consistent with normal physiological parameters, and large numbers of goblet cells were present. However, with increasing salinity, the number of intestinal villi decreased, villus rupture and hyperplasia occurred, and the intestinal mucosa detached from the underlying muscularis layer (Figure 1).

In the control group (0 ppt), the liver cell structure of the juvenile frogs was clear, with cells arranged in a well-organized network, exhibiting normal elliptical or polygonal shapes. The nuclei were centrally located and had well-defined boundaries. In contrast, in the groups exposed to varying salinity levels, bleeding spots appeared in the liver tissue, the liver cell cords widened, the cellular arrangement became disordered, and the cell boundaries became indistinct. The nuclei shifted from their central position, and vacuoles formed within the cells. As the salinity concentration increased, the number of bleeding spots and vacuoles increased, and the extent of liver tissue damage progressively worsened (Figure 1).

### 3.3. Effects of Salinity on the Hepatic Glucose and Lipid Metabolism of Juvenile Bullfrogs

**Figure 2:**
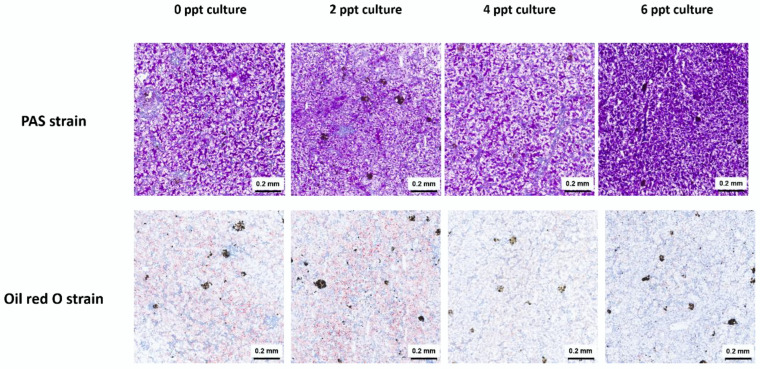
Effects of salinity on the hepatic glucose and lipid metabolism of juvenile bullfrogs (*A. catesbeiana*).

After periodic acid–Schiff (PAS) staining, microscopic examination revealed that the glycogen in the livers of control group frogs (0 ppt) showed a characteristic purple-red reaction, with glycogen granules evenly distributed throughout the liver cytoplasm. Following culture at varying salinity levels, the color characteristics of glycogen in liver cells were comparable to those in the control group. A substantial number of PAS-positive particles remained visible under the microscope and were uniformly distributed in the cytoplasm (Figure 2).

Liver lipids appeared red following Oil Red O staining. The livers of the control group (0 ppt) showed abundant, evenly distributed lipid deposition. After exposure to 2 ppt salinity, liver lipid deposition showed no significant changes compared to the control group. However, at salinities of 4 and 6 ppt, a significant reduction in liver lipid deposition was observed in the juvenile bullfrogs (Figure 2).

### 3.4. Effects of Salinity on the Digestive Enzyme Activities of Juvenile Bullfrogs

**Figure 3:**
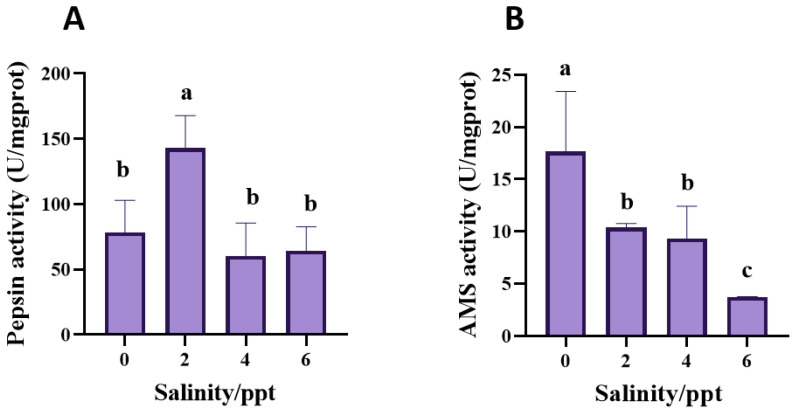
Effects of salinity on the digestive enzyme activities of juvenile bullfrogs (*A. catesbeiana*). (**A**) pepsin activity; (**B**) Amylase (AMS) activity. The small letters on the column indicate significant differences between different salinity groups (*p* < 0.05).

Figure 3 illustrates the effects of different salinity levels (0, 2, 4, and 6 ppt) on pepsin and amylase activities in juvenile bullfrogs. In the 2 ppt salinity group, pepsin activity was significantly higher compared to other salinity groups (*p* = 0.001, Figure 3A). Amylase activity in the control group was significantly higher than in the other salinity groups, with the 6 ppt salinity group showing the lowest activity (*p* = 0.001, Figure 3B).

### 3.5. Effects of Salinity on the Serum Biochemical Indices of Juvenile Bullfrogs

**Figure 4:**
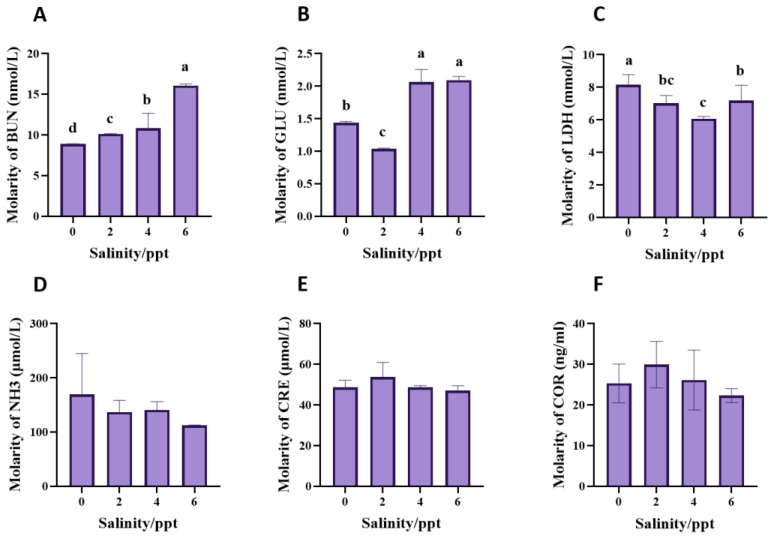
Effects of salinity on the serum biochemical indices of juvenile bullfrogs (*A. catesbeiana*). (**A**) Blood urea nitrogen (BUN), (**B**) Glucose (GLU), (**C**) Lactate dehydrogenase (LDH), (**D**) NH_3_, (**E**) Creatinine (CRE), (**F**) Cortisol (COR). The small letters on the column indicate significant differences between different salinity groups (*p* < 0.05).

Blood urea nitrogen (BUN) levels increased progressively with increasing salinity concentrations, with the highest value observed at 6 ppt (*p* < 0.0001, Figure 4A). In the 4 and 6 ppt groups, glucose (GLU) levels were elevated, with no statistically significant difference between these two groups (*p* = 0.685). In contrast, glucose levels at 0 and 2 ppt were significantly lower, with the lowest levels recorded at 2 ppt (*p* < 0.0001, Figure 4B). Lactate dehydrogenase (LDH) levels decreased significantly in the 2 and 4 ppt salinity groups (*p* = 0.004), while in the 6 ppt salinity group, LDH levels increased but remained below those in the 0 ppt salinity group (Figure 4C). Conversely, NH_3_, creatinine (CRE), and cortisol (COR) levels exhibited no significant differences among the salinity groups and the control group (*p* (NH_3_) = 0.293, *p* (CRE) = 0.177, *p* (COR) = 0.291, Figure 4D–F).

### 3.6. Effects of Salinity on the Antioxidant Enzymes Activities of Juvenile Bullfrogs

**Figure 5:**
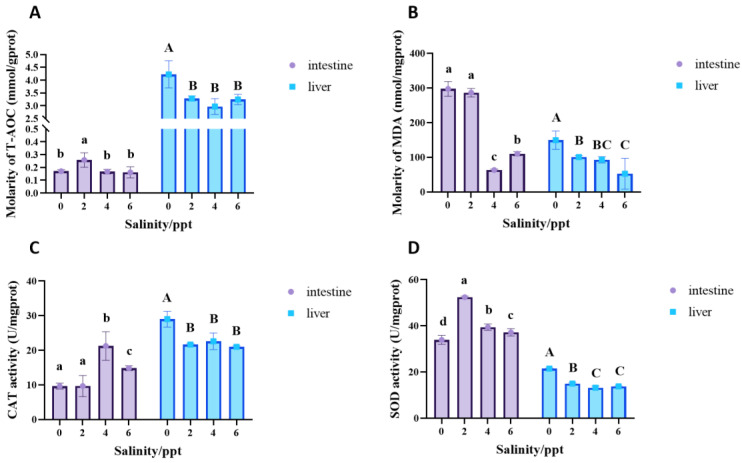
Effects of salinity on the antioxidant enzymes activities of juvenile bullfrogs (*A. catesbeiana*). (**A**) Total antioxidant capacity (T-AOC), (**B**) Malondialdehyde (MDA), (**C**) Catalase (CAT), (**D**) Superoxide dismutase (SOD). The small letters and the capital letters on the column indicate significant differences between different salinity groups in the intestine and liver, respectively (*p* < 0.05).

Figure 5 illustrates the effects of varying salinity levels (0, 2, 4, and 6 ppt) on antioxidant enzyme activities in the livers and intestines of the juvenile bullfrogs. In the intestinal tissue, total antioxidant capacity (T-AOC) levels in the 2 ppt group were significantly higher than those in both the control group and other salinity groups (*p* =0.01, Figure 5A). In contrast, the control group (0 ppt) exhibited the highest T-AOC levels in the liver. The control group also demonstrated elevated malondialdehyde (MDA) levels in the intestine, which significantly decreased at salinities of 4 and 6 ppt (*p* < 0.0001). The liver showed a similar trend, with the lowest MDA concentrations observed at the highest salinity levels (Figure 5B). In the intestinal tissue, catalase (CAT) levels in the 4 and 6 ppt groups were significantly higher than those in the 2 ppt group and the control group (*p* < 0.0001, Figure 5C), while the control group (0 ppt) had the highest CAT levels in the liver. The intestine showed a significant increase in superoxide dismutase (SOD) activity at salinities of 2, 4, and 6 ppt compared to the control group, with the 2 ppt group showing the highest SOD activity. In contrast, the liver’s SOD activity remained relatively stable across salinity levels, with a significant reduction observed in the 2, 4, and 6 ppt salinity groups (*p* < 0.0001, Figure 5D).

## 4. Discussion

Understanding the salinity tolerance of juvenile bullfrogs provides critical insights into how amphibians may cope with increasing salinization in freshwater ecosystems [2]. Salinization, driven by climate change, agricultural runoff, and urbanization, poses a significant threat to the survival and growth of many aquatic organisms, including amphibians [3,4]. Amphibians are particularly sensitive to changes in salinity due to their permeable skin and limited osmoregulatory capacity, making them highly susceptible to osmotic stress. This study provides significant insights into how juvenile bullfrogs respond to salinity stress, which can help forecast their resilience to environmental changes, both in aquaculture settings and in natural habitats.

The results of this study show that juvenile bullfrogs exhibit moderate resilience to low and moderate salinity levels (2–4 ppt), with no significant changes in survival rates. However, at higher salinity levels (6 ppt), physiological indicators such as digestive enzyme activity, antioxidant enzyme levels, and histological changes in the liver and intestine reveal that salinity stress negatively affects critical organ function. These findings align with previous research on other amphibian species, which similarly found that elevated salinity levels disrupt normal physiological processes and reduce overall fitness. Research on adult frog of *Pelophylax nigromaculatus* has shown that increased salinity levels not only reduce growth rates but also impair metabolic functions and increase oxidative stress, ultimately compromising the frog’s ability to survive and develop [16,17].

Nutrients are digested by digestive enzymes such as trypsin, lipase, amylase and pepsin, and it has been confirmed that acute salinity stress can affect the enzymes’ activity [21]. Digestive enzymes are directly involved in nutrient digestion and absorption, which are critical for maintaining energy balance and growth under stress [22]. Salinity stress can alter enzymatic activity by affecting the biochemical environment of the gut, potentially reducing digestive efficiency and nutrient assimilation [21,22]. Monitoring these enzymes provides insights into how salinity influences metabolic processes critical for growth and survival. In terms of digestive function, the study identified that pepsin and amylase activities in juvenile bullfrogs were most efficient at 2 ppt salinity, suggesting that moderate salinity may enhance digestive enzyme activity, improving nutrient absorption and growth. However, at 6 ppt, both enzyme activities were significantly reduced, indicating impaired digestive capacity. Similar reductions in digestive efficiency have been observed in other aquatic species, such as oysters (*Crassostrea hongkongensis*) [23] and spotbanded scats (*Selenotoca multifasciata*) [24] in which high salinity levels reduce enzyme activity and growth performance. The decline in enzyme function at higher salinities could explain why juvenile bullfrogs experience stunted growth and higher susceptibility to disease under these conditions [7,25].

Enzymes such as superoxide dismutase (SOD) and catalase (CAT) play a vital role in mitigating oxidative stress caused by reactive oxygen species (ROS) [26]. Salinity-induced osmotic stress can disrupt cellular homeostasis, leading to increased ROS production [27]. Antioxidant enzyme activity serves as a proxy for the organism’s ability to counteract oxidative damage, which is essential for maintaining cell integrity and function under saline conditions [28]. The study also highlighted the oxidative stress experienced by juvenile bullfrogs at higher salinity levels. Increased malondialdehyde (MDA) levels and decreased antioxidant enzyme activity (SOD and CAT) were clear indicators of lipid peroxidation and oxidative damage to cells [29,30]. This finding is consistent with other research on amphibians, such as the *Xenopus laevis* [29], which have demonstrated that prolonged exposure to saline conditions induces oxidative stress, leading to cell damage and reduced organ function. This oxidative stress could have long-term implications for the health and survival of bullfrogs in increasingly saline environments.

While the findings of this study provide important insights, there are limitations that must be acknowledged. First, the study only examined short-term exposure (one week) to salinity stress. The long-term effects of salinity exposure on juvenile bullfrogs, including potential impacts on reproductive success and adult survival, remain unexplored. Additionally, while this study focused on juvenile bullfrogs, future research should investigate how frogs at different life stages—particularly adults—respond to salinity stress. Finally, it is crucial to explore the molecular mechanisms underlying salinity tolerance, particularly the genes involved in osmoregulation and oxidative stress response, to better understand the adaptive capacity of bullfrogs and other amphibians to saline environments.

## 5. Conclusions

In conclusion, this study provides critical insights into the salinity tolerance of juvenile bullfrogs, laying the foundation for understanding how amphibians may adapt to the salinization of freshwater ecosystems. Amid ongoing environmental changes driven by climate change and human activities, understanding the physiological responses of amphibians to salinity stress is essential for both conservation efforts and the development of sustainable aquaculture practices.

## Figures and Tables

**Table 1:** Effect of salinity on the survival of juvenile bullfrogs (*Aquarana catesbeiana*).

Salinity (ppt)	Survival Rate (%)
0	100
2	100
4	100
6	100

## Data Availability

Data and materials will be available on request.
